# Identification and analysis of metabolite production with biotechnological potential in *Xanthophyllomyces dendrorhous* isolates

**DOI:** 10.1007/s11274-015-1808-3

**Published:** 2015-02-03

**Authors:** Gabriela Contreras, Salvador Barahona, Dionisia Sepúlveda, Marcelo Baeza, Víctor Cifuentes, Jennifer Alcaíno

**Affiliations:** Departamento de Ciencias Ecológicas, Facultad de Ciencias, Universidad de Chile, Las Palmeras 3425, Casilla 653, Ñuñoa, Santiago Chile

**Keywords:** Carotenoids, Astaxanthin, *Xanthophyllomyces dendrorhous*, *Phaffia rhodozyma*, Polyunsaturated fatty acids

## Abstract

Antarctic microorganisms have developed different strategies to live in their environments, including modifications to their membrane components to regulate fluidity and the production of photoprotective metabolites such as carotenoids. Three yeast colonies (ANCH01, ANCH06 and ANCH08) were isolated from soil samples collected at King George Island, which according to their rDNA sequence analyses, were determined to be *Xanthophyllomyces dendrorhous*. This yeast is of biotechnological interest, because it can synthesize astaxanthin as its main carotenoid, which is a powerful antioxidant pigment used in aquaculture. Then, the aim of this work was to characterize the ANCH isolates at their molecular and phenotypic level. The isolates did not display any differences in their rDNA and *COX1* gene nucleotide sequences. However, ANCH01 produces approximately sixfold more astaxanthin than other wild type strains. Moreover, even though ANCH06 and ANCH08 produce astaxanthin, their main carotenoid was β-carotene. In contrast to other *X. dendrorhous* strains, the ANCH isolates did not produce mycosporines. Finally, the ANCH isolates had a higher proportion of polyunsaturated fatty acids than other wild type strains. In conclusion, the reported *X. dendrorhous* isolates are phenotypically different from other wild type strains, including characteristics that could make them more resistant and better able to inhabit their original habitat, which may also have biotechnological potential.

**Electronic supplementary material:**

The online version of this article (doi:10.1007/s11274-015-1808-3) contains supplementary material, which is available to authorized users.

## Introduction

Antarctica is one of the most physically and chemically extreme environments on Earth, of extreme cold and a high incidence of solar radiation with an elevated ultraviolet B (UV-B) light component; however, a variety of fungi (including yeasts) have been isolated or detected in this territory. Psychrophilic and psychro tolerant microorganisms must overcome the challenges that arise because of the low temperature, which affects the rate of biochemical reactions and the viscosity of their environment (D’Amico et al. 2006). Changes in lipid metabolism are known to constitute a major adaptation to improve membrane fluidity and to maintain its optimal functionality (Rossi et al. 2009). In this sense, one important adaptation that helps to maintain fluidity at low temperatures is the degree of unsaturation of the membrane fatty acids (FAs) (Dexter and Cooke 1984). In general, at lower growth temperatures, a higher content of monounsaturated (MUFA), polyunsaturated (PUFA) and methyl-branched FAs are produced (Amaretti et al. 2010; D’Amico et al. 2006). This is an important point because the ingestion of PUFAs, specifically, on a diet with a high omega-3/omega-6 ratio, has significant effects on human health, including the prevention of inflammatory, autoimmune and cardiovascular diseases (Simopoulos 2002). Sterols are also essential structural and regulatory components of eukaryotic cell membranes that affect their fluidity (Dexter and Cooke 1984), and ergosterol is the main sterol produced by yeasts (Nes et al. 1978). The importance of ergosterol in stress response has been suggested, promoting resistance to freeze-thaw and cold-shock stresses (Calcott and Rose 1982), to high-pressure conditions and low temperatures (Abe and Minegishi 2008). This sterol is also an economically important metabolite because it is the precursor of Vitamin D2 (Veen and Lang 2004), which is used for anti-rachitic treatment and in food fortification (Vandamme 1992).

Microorganisms have developed multiple photoprotectives mechanisms for protection against UV radiation, including synthesis of molecules that are able to absorb UV radiation directly, such as mycosporines, and/or antioxidants that decrease the stress caused by radiation, such as carotenoids (Roy 2000). Carotenoids are natural yellow, orange and red pigments that provide protection against photooxidative damage and in recent decades, an increasing number of reports have described the beneficial effects of carotenoids on animal and human health. Among carotenoids, astaxanthin (3,3′-dihydroxy-β,β-carotene-4-4′-dione) stands out for its antioxidant properties, which have been reported to be greater than those of β-carotene and even α-tocopherol (Miki 1991). For these reasons, the application of astaxanthin in the pharmaceutical and cosmetic industries has been explored (Higuera-Ciapara et al. 2006). Conversely, astaxanthin has been widely used in the aquaculture industry as a colorant for cultured salmonids to achieve the flesh color that is desired by consumers.

Astaxanthin production is limited to a few organisms, including the yeast *Xanthophyllomyces dendrorhous* (asexual stage: *Phaffia rhodozyma*). However, the production of this pigment by wild-type strains is too low (200–400 μg g^−1^ of dry yeast) to provide a natural source that is economically competitive. *X. dendrorhous* is a moderate psychrophilic basidiomycete that most likely evolved in a cold climate (Ducrey Sanpietro and Kula 1998). This yeast was originally isolated from slime exudates of deciduous trees in mountainous regions of Alaska and Japan (Phaff et al. 1972) and other isolates were later obtained from cold areas of Russia and Finland (Golubev 1995). More recently, *X. dendrorhous* strains were obtained from Italy, Germany (Weber et al. 2006), the United States (Fell et al. 2007), the Argentinean Patagonia (Libkind et al. 2007) and Chile (Weber et al. 2008). It is noteworthy that all of the original habitats of the *X. dendrorhous* isolates share stressful environmental conditions. These stressful conditions include high UV exposure (Libkind et al. 2011) and/or oxidative stress produced by the antimicrobials and antiparasitic compounds synthesized by the host tree, which generate reactive oxygen species (Schroeder and Johnson 1993, 1995). In addition to carotenoids, it has been reported that *X. dendrorhous* can synthesize other economically important secondary metabolites such as mycosporines (Libkind et al. 2011).

This work studied *X. dendrorhous* strains that were isolated from soil samples collected from terrestrial habitats on King George Island, which is the major island of the Shetland South Archipelago in the Antarctic Peninsula (Carrasco et al. 2012). The isolates were molecularly characterized based on their rDNA nucleotide sequences and using the micro/minisatellite primed-PCR (MSP-PCR). Additionally, mitochondrial cytochrome *c* oxidase subunit 1 (*COX1*) genes nucleotide sequences were evaluated. The *COX1* gene sequence that encodes amino acids 19-234 has been used for animal identification (DNA barcoding) (Hebert et al. 2003) and recently, fungal intra-species variability in this gene has been reported in strains from the genera *Penicillium* (Seifert et al. 2007) and *Leohumicola* (Nguyen and Seifert 2008). In addition, the production of metabolites with biotechnological potential, such as carotenoids, ergosterol, PUFAs and mycosporines was evaluated, with promising results.

## Methods and methods

### Yeast isolation, microorganisms and culture conditions

Thirty-four soil samples were collected from King George Island, Antarctic Peninsula, on January 2010. The samples were processed as described by Carrasco et al. (2012) for yeast isolation on MYP media plates [0.7 % malt extract, 0.05 % yeast extract, 0.25 % peptone-soytone, and 2.0 % agar (pH 5.0)] (Libkind et al. 2007) that were supplemented with 25 µg mL^−1^ chloramphenicol. According to the phenotype, as determined by microscopic examination, yeast colonies similar to *X. dendrorhous* were recovered, immediately transferred to fresh MYP plates and incubated at 22 °C.

Thirteen *X. dendrorhous*-like colonies were isolated from a soil sample collected on the Barton peninsula (62° 14.074′ WO 58° 46.567′), which were named ANCH01 to 13 and preserved by the dehydrated gelatin drop method (Baeza et al. 2009). The *X. dendrorhous* wild type strains UCD 67-385 (ATCC 24230), which was isolated from a *Betula tauschii* tree in Kiso, Japan, and AVHN2 (Loto et al. 2012) which was isolated from *Gevuina avellana* leaves in the Biobío region of Chile, were included in this study for comparative purposes. Yeasts were grown at 22 °C with constant agitation in YM media (1 % glucose, 0.3 % yeast extract, 0.3 % malt extract and 0.5 % peptone) or YM-agar plates (1.5 % agar). The calculated generation time (*g*) was calculated according Madigan et al. (1997), it is corresponded to the average of three independent cultures incubated at 22 °C in YM medium with constant agitation.

The ability of the ANCH isolates to develop sexual structures (basidia with basidiospores) was evaluated on distilled water ribitol medium (DWR) agar plates (0.5 % ribitol) (Kucsera et al. 1998) and on vogel minimal medium (MMv) agar plates that were supplemented with 2 % glucose (Retamales et al. 2002). Strains were initially incubated for 3 days at 22 °C and then transferred to 10 °C until the sexual structures emerged.

### UV-B survival experiments

*X. dendrorhous* strains were grown at 22 °C with constant agitation in YM media until reaching the early-stationary phase (DO_600_ 10 to 11) and cells were harvested by centrifugation at 4,000g for 5 min. The cell pellets were washed, suspended in sterile distilled water to reach a final concentration of approximately 1 × 10^7^ cells mL^−1^ and transferred to sterile plates to be exposed to UV-B radiation (310 nm) at 6.76 mW cm^−2^, using a lamp with a Phillips 20 W F20T/12 UV-B tube. Samples of 0.5 µL were taken after 0, 0.5, 1, 2 and 3 h of UV-B exposure (12,177; 24,354; 48,708 y 73,062 mJ cm^−2^, UV-B doses respectively), and the samples were diluted and seeded onto YM-agar plates to obtain isolated colonies. The UV-B tolerance was evaluated as the survival percentage, and the results represent the average of three independent experiments for each strain.

### DNA amplification and MSP-PCR fingerprinting

*X. dendrorhous* DNA was extracted from protoplasts as described previously (Hermosilla et al. 1995) from yeast cultures grown in YM media at 22 °C with constant agitation. Three regions of the nuclear rDNA were analyzed: the D1/D2 domain of the large ribosomal subunit (26S) rDNA (Scorzetti et al. 2002), the ITS (Internal Transcribed Spacer) region, which included ITS1, the 5.8S gene and ITS2 (Scorzetti et al. 2002), and the IGS1 (Intergenic Transcribed Spacer 1) region, which extends from 26S to the 5S gene (Sugita et al. 2002). The PCR-amplification and sequencing of the D1/D2 and ITS regions were performed using the primer pair ITS5 (5′-GGAAGTAAAAGTCGTAACAAGG-3′) and LR6 (5′-CGCCAGTTCTGCTTACC-3′) (White et al. 1990), while the IGS1 region with LR12 (5′-GACTTAGAGGCGTTCAG-3′) and 5SRNA (5′-ATCAGACGGGATGCGGT-3′) (Vilgalys and Gonzalez 1990). The obtained nucleotide sequences were uploaded to the GenBank database: the ANCH01, 06 and 08 large-subunit rDNA D1/D2 domains sequences [KF731820, KF731821 and KF731822, respectively], the ITS region [KF731816, KF731817 and KF731818, respectively] and the IGS region [KF731824, KF731825 and KF731826, respectively]. For amplification and sequence analysis of the partial *X. dendrorhous* mitochondrial cytochrome c oxidase subunit 1 (*COX1*) gene, the COX1.5.F (5′-CAGAAAAGGTGCTGGTACAGC-3′) and COX1.7.R (5′-TACTGCCTTACGGCAATCTG-3′) primers were designed and used in this study. All oligonucleotides were synthesized and purchased from Alpha DNA (Canada) or from Integrated DNA Technologies (USA). The PCR reactions were performed according to Loto et al. (2012) in a 2720 Applied Biosystems thermal cycler and the amplification products were separated by 0.8 % agarose gel electrophoresis in TAE buffer stained with ethidium bromide (Sambrook and Russell 2001) and then recovered form gels as previously described (Boyle and Lew 1995). The 100 bp and 1 kb DNA Ladder were used as molecular weight standards. Nucleotide sequences were determined using the ET terminator Kit from Amersham Bioscience (Piscataway, New Jersey, USA) with an ABI 3100 Avant Genetic Analyzer (Applied Biosystems) and analyzed using the Bioedit (http://www.mbio.ncsu.edu/bioedit/bioedit.html) and BLAST programs (NCBI). For phylogenetic analysis, the MEGA version 5.2 software (Tamura et al. 2011) was used with maximum-parsimony method and 1,000 bootstrap replicates.

For the MSP-PCR fingerprinting experiments, the synthetic oligonucleotides (GTG)_5_ and (GAC)_5_ were employed and the PCR reactions were performed as previously described (Libkind et al. 2007). The amplified DNA fragments were separated by gel electrophoresis on 1.4 % agarose gels in TAE buffer, stained with ethidium bromide and visualized under a UV transilluminator.

### Fatty acid composition analysis

Biomass for the FA analyses was obtained from 1 L of 120-h-old yeast cultures grown at 22 °C with constant agitation in YM media. The cell pellets were harvested by 5 min of centrifugation at 4,000 g and the pellets were thoroughly washed with distilled water. Oil extraction was conducted according the method described by Bligh and Dyer (1959) and the FA composition analysis was determined by gas chromatography. Oil extraction and FA composition analysis was performed by an external service at GCL, *Fundación Chile* (http://www.eurofins.cl/).

### Sterol, carotenoids and mycosporine extraction and analysis

Sterol, carotenoids and mycosporines were extracted from cellular pellets from 120-h-old yeast cultures (stationary phase of growth), which had been incubated at 22 °C with constant agitation in YM media. Sterols were extracted with of petroleum ether after saponification at 80 ± 2 °C for 2 h of cell pellets with KOH and 60 % (v/v) ethanol solution (Shang et al. 2006) and quantified based on the 282 nm absorbance values with the molar extinction coefficient of 11,900 M^−1^ cm^−1^, according to Venkateswarlu et al. (1998). Carotenoids were extracted using the acetone extraction method (An et al. 1989) and quantified by absorbance at 465 nm using an absorption coefficient of A1 % = 2,100. Mycosporines were extracted with a 20 % (v/v) methanolic aqueous solution, following Libkind et al. (2005) and quantified at 310 nm and the molar extinction coefficient of mycosporine-glutaminol-glucoside (25,000 M^−1 ^cm^−1^), according to previous reports (Bouillant et al. 1981). Sterols, carotenoids and mycosporines were dried, suspended in acetone and separated by RP-HPLC using a reverse phase RP-18 Lichrocart 125-4 column (Merck) with methanol:water (97:3, v/v), acetonitrile:methanol:isopropanol (85:10:5, v/v) or water:methanol:acetic acid (993:5:2, v/v), respectively, as the mobile phase with a 1 mL min^−1^ flux under isocratic conditions. The elusion spectra were recuperated using a diode array detector, and metabolites were identified by their spectra and retention time according to standards. The analyses were performed in triplicate, and metabolite production was normalized relative to the dry weight of the yeast.

### Mutagenesis

Random mutagenesis was performed using *N*-methyl-*N*’-nitro-*N*-nitrosoguanidine (NTG) at a final concentration of 50, 80, 100, 120 and 150 μg mL^−1^ according to Retamales et al. (1998). For gamma ray irradiation, cells were treated according to Najafi et al. (2011) and an external service was hired for irradiation at the *Comisión Chilena de Energía Nuclear* (http://www.cchen.cl/) using 0.5, 1.0, 1.5, 2.0, 2.5, 3.0, 3.5, 5.0 and 6.0 kGy doses.

## Results

### Yeast isolation and general characterization

**Fig. 1:**
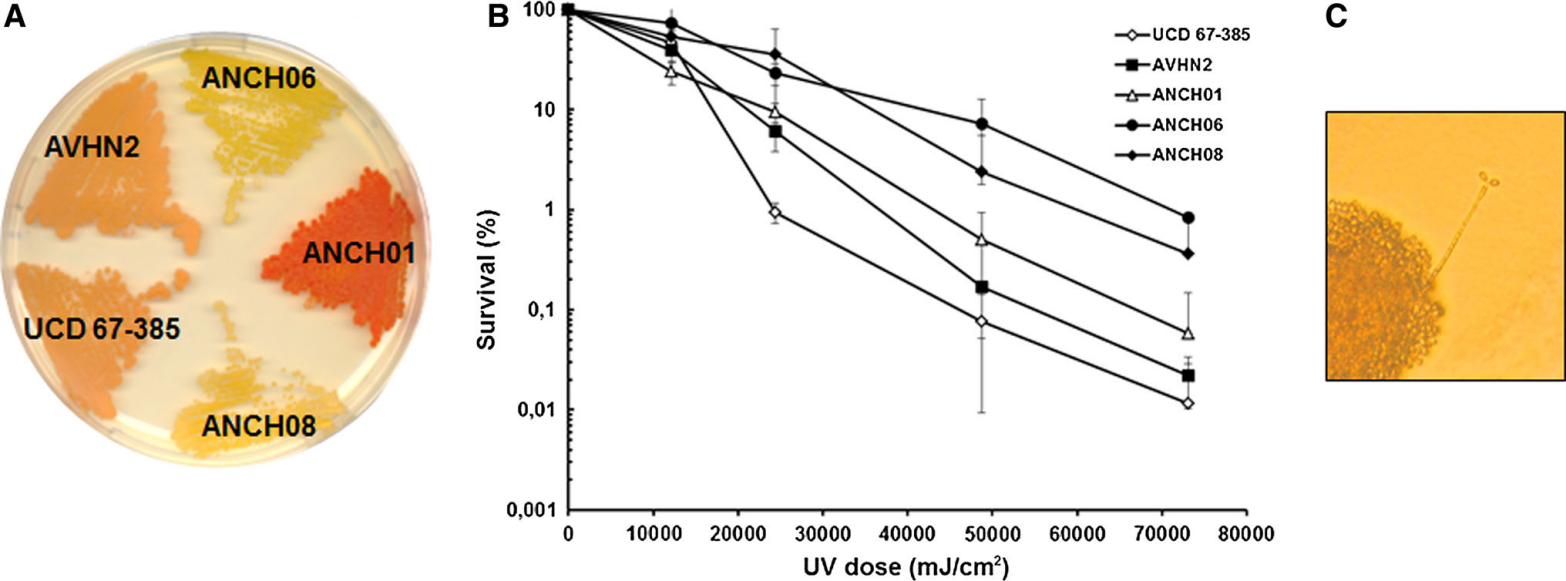
Phenotype and survival after UV-B irradiation of *X. dendrorhous* strains and King George Island isolates. The color phenotypes and survival percentages after exposure to UV-B (310 nm) radiation of ANCH01, ANCH06 and ANCH08 were compared to AVHN2 and UCD 67-385 wild type strains. **a** Strains cultivated on YM-agar plates for 4 days at 22 °C. **b** Survival percentage following of 73,062 mJ cm^−2^ UV-B dose (3 h of exposure) (average of three independent cultures). **c** Holobasidium with terminal basidiospores of ANCH08, after 4 days of culture at 22 °C and then 23 days at 10 °C on DWR medium

Thirteen yeast colonies that shared a similar macromorphological phenotype (colony shape, texture and color) to *X. dendrorhous* strains were isolated from one Antarctic soil sample that was incubated at 22 °C. Three color phenotypes could be distinguished among the isolates: red, pale-yellow and yellow, then one representative isolate from each group was randomly chosen for further analyses and named ANCH01, ANCH06 and ANCH08, respectively (Fig. 1a). The growth kinetics of these isolates was evaluated at 22 °C because this is the optimal growth temperature that has been reported for *X. dendrorhous* (An et al. 1989). The generation times were 11.12 ± 0.05, 8.44 ± 0.71 and 8.94 ± 0.28 h for ANCH01, ANCH06 and ANCH08, respectively.

Considering the original habitat of the ANCH isolates, the UV-B radiation tolerance at 310 nm was evaluated and compared to the wild type strains AVHN2 and UCD 67-385 that were isolated from other geographical regions (Fig. 1b). ANCH06 had a higher survival rate at UV-B dose of 73,062 mJ cm^−2^ (3 h of exposure) that was approximately 1.5-fold higher than UCD 67-385 and AVHN2, which was statistically significant according to Student’s t test (*p* < 0.05).

### Molecular characterization

To confirm the identity of the ANCH isolates, total DNA was extracted to PCR-amplify and sequence the D1/D2 domains of the large-subunit rDNA and the ITS rDNA region. The D1/D2 and ITS sequences of the ANCH isolates and the AVHN2 strain were identical and the BLASTn analysis of the D1/D2 consensus nucleotide sequences from the ANCH isolates in the Genbank database, the best hit was *P. rhodozyma* CBS5905 [GenBank: AF189871.2] with 100 % identity. Also, a dendrogram was constructed based on the *X. dendrorhous*/*P. rhodozyma* ITS sequences used in other works (Libkind et al. 2007; Weber et al. 2008), which showed that the ANCH isolates belong to the cluster that includes strains isolated from the Argentinean Patagonia (Supplementary Figure 1). These results indicate that the ANCH isolates indeed correspond to *X. dendrorhous*/*P. rhodozyma* strains. Currently, the anamorphic strains are designated as *P. rhodozyma* and the teleomorphic strains as *X. dendrorhous* (Golubev 1995). Then, the ability of the ANCH isolates to develop sexual structures was also evaluated. ANCH01 developed basidia with basidiospores after 1 month of incubation in MMv that was supplemented with 2 % glucose and on DWR medium at 10 °C. ANCH06 and ANCH08 developed these structures after only 2 weeks of incubation at 10 °C in both tested media (Fig. 1c). Therefore, these isolates were classified as *X. dendrorhous*.

To evaluate the variability among the ANCH isolates, the IGS1 rDNA region was amplified. The IGS1 sequence among the ANCH isolates and the AVHN2 strain [GenBank: KF731827] were identical, sharing 80.7 % identity with the UCD 67-385 strain. The best IGS1 GenBank BLASTn hit (*X. dendrorhous* CRUB0853 [GenBank: DQ661032.1]) shared 86 % identity with the ANCH isolates at this region. Similarly, the ANCH isolates and the AVHN2 strain MSP-PCR fingerprinting analyses resulted in identical amplification patterns, but these patterns were different from that of the UCD 67-385 strain (data not shown). Furthermore, the variability of the ANCH isolates was evaluated by determining the sequence of *COX1* gene. Using our *X. dendrorhous* genomic database from strain UCD 67-385, were able to identify the putative *COX1* gene [GenBank: KF731815], which comprises 1,665 bp with no intronic sequences and encodes a predicted protein of 554 amino acids. From its genomic sequence, specific primers to amplify the DNA barcode region were designed, revealing that it is identical among the ANCH isolates but it has 99.8 and 98.3 % identity with strains AVHN2 and UCD 67-385, respectively. Therefore, it was not possible to find intra-species variability among the ANCH isolates using this strategy, but they were differentiated from AVHN2. Then, the molecular marker analyses of the yeast isolates studied in this work indicate that the ANCH isolates are more similar and closely related to AVHN2 than to UCD 67-385, most likely due to their different geographic origins.

### Metabolite production analysis

#### Mycosporine production

The ANCH isolates did not produce mycosporines, at least under the conditions in which the assay was performed. However, under the same conditions, AVHN2 and UCD 67-385 produced 45.59 ± 1.87 and 3.67 ± 2.51 mg g^−1^ dry yeast, respectively, of a single type of mycosporine. This product was identified as mycosporine-glutaminol-glucoside, according to the results obtained using other *X. dendrorhous* strains (Libkind et al. 2011).

#### Lipid production

The results of lipid production are summarized in Table 1. As expected, the main sterol produced by all of the analyzed strains was ergosterol (94–100 %). However, no significant differences were observed in the ergosterol content, except for ANCH06. ANCH06 produced approximately threefold less ergosterol than the other assayed strains under the same conditions. Regarding the composition of the FAs, linoleic, oleic and palmitic acids were observed in all of the assayed strains. The ANCH isolates have a similar proportion of PUFAs in relation to total FAs, which is higher than the value observed in AVHN2 and twice the value observed in UCD 67-385. This difference is mainly due to linolenic acid abundance.

**Table 1:** Ergosterol production and fatty acids composition in ANCH isolates and the AVHN2 and UCD 67-385 strains

Strain/ isolate	Ergosterol (mg g^−1^ dry yeast)^a^	Percentage of total fatty acids															
		Saturated								Monounsaturated					Polyunsaturated		
		<c_14:0_	Myristic acid c_14:0_	c_15:0_	Palmitic acid c_16_	c_17_	Stearic acid c_18_	>c_18_	Total	Palmitoleic acid c_16:01_	c_17:01_	Oleic acid c_18:1_	c_20:01_	Total	Linoleic acid c_18:2_ (ω-6)	α-linolenic acid c_18:3_ (ω-3)	Total
ANCH01	3.52 ± 0.55 (95.83 ± 1.92)	ND	0.24	ND	14.27	1.71	7.42	0.35	**23.99**	0.59	ND	41.65	ND	**42.17**	30.49	3.35	**33.84**
ANCH06	0.99 ± 0.80 (100)	0.06	0.85	0.36	23.11	0.32	6.67	1.25	**32.62**	0.49	0.04	29.31	0.09	**29.93**	35.52	1.94	**37.46**
ANCH08	4.21 ± 0.92 (97.47 ± 1.66)	ND	0.8	0.34	22.95	0.83	7.94	1.27	**34.13**	0.33	0.15	29.04	0.07	**32.02**	32.1	1.75	**33.85**
AVHN2	3.34 ± 0.10 (98.54 ± 0.69)	0.17	0.64	0.28	32.1	0.65	21.75	0.89	**56.48**	ND	ND	15.99	ND	**16.37**	23.92	3.25	**27.17**
UCD 67-385	3.65 ± 0.82 (99.22 ± 0.69)	0.05	0.45	0.14	10.20	0.18	6.75	1.49	**19.26**	0.52	ND	65.16	0.15	**65.83**	12.56	2.35	**14.91**

Total values of metabolites of each type are shown in bold

*ND* not detected, *C*_*N*:*D*_ “N” represents the number of carbon atoms of each FA, and “D” is the number of double bonds in the fatty acids

^a^Table values are the average results of three independent experiments ± standard deviation. Ergosterol percentage relative to total sterols is indicated in parentheses

#### Carotenoid production

The carotenoid production of the ANCH isolates was evaluated after 120 h of cultivation in YM media at 22 °C with constant agitation. These results were compared to the production of the AVHN2 and UCD-67-385 strains, and the data are summarized in Table 2. ANCH01 produces the highest amount of total carotenoids, approximately sixfold more than the other strains analyzed. Moreover, astaxanthin is the main carotenoid accumulated in this isolate, representing approximately 70 % of the total carotenoid content. Astaxanthin reached approximately 900 µg g^−1^ dry yeast under the studied conditions, approximately eightfold more than the UCD 67-385 strain. Additionally, ANCH01 produces fivefold more phoenicoxanthin, which is precursor to astaxanthin, than AVHN2 and UCD 67-385. Moreover, the main carotenoid accumulated by the yellow isolates ANCH06 and ANCH08 was β-carotene instead of astaxanthin. However, they produced a minor fraction of astaxanthin. For this reason, the carotenogenic genes [reviewed in Schmidt et al. (2011)] of one yellow isolate, ANCH08, were sequenced and compared to the ones in the red isolate ANCH01. Among them, the *idi*, *FPS*, *crtE*, *crtYB*, *crtI*, *crtS* and *crtR* were included and no differences were found between the corresponding genes from ANCH01 and ANCH08 [GenBank: KM496268, KM496269, KM496270, KM496271, KM496272, KM496273 and KM496274: ANCH08 *idi*, *FPS*, *crtE*, *crtYB*, *crtI*, *crtS* and *crtR* genes, respectively]. Considering this result, it is expected that the astaxanthin production in the ANCH08 isolate can be enhanced by random mutagenesis. So, this isolate was submitted to NTG (Retamales et al. 1998) and gamma irradiation (Najafi et al. 2013) treatments. In this way, several mutants with a reddish hue were obtained: ten by 150 μg mL^−1^ NTG treatment and four with 5 kGy of gamma irradiation. One mutant from each treatment (named ANCH08-NTG150-A and ANCH08-gamma5-A, respectively) was randomly chosen to evaluate carotenoid production. Both mutants and the parental strain were cultured in parallel at 22 °C in YM media with constant agitation, and after 5 days of cultivation, carotenoids were extracted. It was determined that the specific carotenoid production (µg carotenoids g^−1^ dry yeast) increased about 1.6-fold in ANCH08-NTG150-A and decreased about 0.8-fold in ANCH08-gamma5-A (Table 2). However, in both mutant strains the astaxanthin fraction increased from 9.45 ± 0.06 % in the parental strain to 26.82 ± 1.37 % and 17.33 ± 2.66 % in ANCH08-NTG150-A and ANCH08-gamma5-A, respectively. This result confirms that indeed the yellow isolates produce astaxanthin and that the production can be favored by random mutagenesis.

**Table 2:** Carotenoid production of ANCH isolates and the AVHN2 and UCD 67-385 strains

Carotenoid (µg g^−1^ dry yeast)	*X. dendrorhous* strains/isolates						
	ANCH01	ANCH06	ANCH08	ANCH08-NTG150-A-1	ANCH08-gamma5-A-1	AVHN2	UCD 67-385
Total carotenoid	1,315.23 ± 217.74 (100)	207.69 ± 6.63 (100)	353.67 ± 28.42 (100)	564.00 ± 64.41 (100)	211.67 ± 9.16 (100)	208.01 ± 81.46 (100)	192.06 ± 11.84 (100)
β-carotene	30.17 ± 15.21 (2.34 ± 1.16)	118.17 ± 12.59 (56.90 ± 6.06)	234.51 ± 18.84 (66.32 ± 1.78)	224.81 ± 29.87 (39.81 ± 1.60)	121.01 ± 8.16 (57.15 ± 2.35)	10.34 ± 0.93 (4.97 ± 0.45)	14.30 ± 1.49 (7.45 ± 0.78)
Echinenone	11.69 ± 1.87 (0.89 ± 0.14)	19.12 ± 3.11 (9.21 ± 1.50)	3.15 ± 1.66 (0.89 ± 0.49)	2.71 ± 1.06 (0.47 ± 0.15)	1.61 ± 0.32 (0.76 ± 0.13)	5.22 ± 0.83 (2.51 ± 0.40)	7.70 ± 1.38 (4.01 ± 0.72)
OH-Echinenone	33.04 ± 4.98 (2.51 ± 3.38)	16.48 ± 5.42 (7.93 ± 2.61)	22.99 ± 4.06 (6.47 ± 0.63)	51.00 ± 7.55 (9.02 ± 0.32)	20.29 ± 2.12 (9.58 ± 0.70)	12.55 ± 6.56 (6.03 ± 3.15)	18.05 ± 1.44 (9.40 ± 0.75)
Phoenicoxanthin	134.54 ± 7.80 (10.23 ± 0.59)	11.31 ± 2.73 (5.44 ± 1.31)	12.13 ± 2.73 (3.41 ± 0.60)	51.54 ± 6.74 (9.14 ± 0.46)	11.53 ± 0.93 (5.45 ± 0.38)	27.36 ± 1.91 (13.15 ± 0.92)	26.63 ± 2.31 (13.86 ± 1.21)
Astaxanthin	988.90 ± 3.91 (75.19 ± 0.3)	29.13 ± 6.76 (14.03 ± 3.26)	33.46 ± 4.26 (9.45 ± 0.80)	150.96 ± 14.26 (26.82 ± 1.04)	36.62 ± 2.73 (17.34 ± 1.73)	130.76 ± 3.43 (62.86 ± 1.65)	110.33 ± 4.11 (57.44 ± 2.14)
Other carotenoids^a^	105.45 ± 10.01 (8.02 ± 0.76)	12.45 ± 6.28 (5.99 ± 3.02)	47.59 ± 4.56 (13.50 ± 1.53)	83.02 ± 7.87 (14.75 ± 0.53)	22.58 ± 2.37 (10.67 ± 1.05)	17.58 ± 4.90 (8.45 ± 2.36)	14.63 ± 1.59 (7.62 ± 0.83)

Table values are the average results of three independent experiments ± standard deviation. Percentage relative to total carotenoids is indicated in parentheses

^a^Other carotenoids include canthaxanthin, OH-keto-torulene, OH-keto-γ-carotene, torulene, neurosporene and unidentified carotenoids

## Discussion

Although *X. dendrorhous* has usually been associated with deciduous trees, it has also been isolated from the parasitic fungus *Cyttaria hariotii* of *Nothofagus* trees, eucalyptus leaves and water samples (Libkind et al. 2007; Weber et al. 2008). However, this work reports the isolation of *X. dendrorhous* from a soil sample, which is an unusual habitat for this yeast. It has been proposed that there is a relationship between the ITS sequence of *X. dendrorhous* isolates and their original tree host, suggesting that there is host specificity in different *X. dendrorhous* strains (Libkind et al. 2007). In support of this idea, it was suggested that *X. dendrorhous* strains isolated from water samples of Lake Ilon in the Argentinean Patagonia were associated with the nearby *Nothofagus* forests (Libkind et al. 2007). This tree genus is distributed in Southern Chile and Argentina, including Cape Horn (Swenson et al. 2001). Therefore, based on the ITS sequence, the most likely original habitats of the ANCH isolates were the *Nothofagus* forests of South America, and the isolates were most likely carried to King George Island by humans (Cowan et al. 2011) and/or high altitude Aeolian processes (Pearce et al. 2009). Based on the ITS sequence analysis, Argentinean strains and the Antarctic *X. dendrorhous* isolates form a genetically uniform and distinct population. However, despite the geographical proximity, these strains do not cluster with the Chilean strain isolated from eucalyptus leaves.

The described *X. dendrorhous* ANCH isolates have several phenotypic differences, such as carotenoid production and UV-B radiation tolerance. Therefore, they may correspond to different *X. dendrorhous* strains. However, the usual molecular techniques used to classify *X. dendrorhous* strains, such as MSP-PCR and rDNA sequence analyses (Libkind et al. 2007), did not differentiate the ANCH isolates in this work. Moreover, the *COX1* gene sequence that was analyzed in this work has been used as a DNA-based identification system in the animal kingdom (Hebert et al. 2003) and, more recently, in the fungal kingdom (López et al. 2003; Nguyen and Seifert 2008; Seifert et al. 2007). Although the *COX1* gene has not been studied before in *X. dendrorhous*, no differences were found in this report between the ANCH isolates. However, differences were observed between the ANCH isolates and the AVHN2 strain, which were not differentiated by rDNA sequence or MSP-PCR analyses.

Mycosporines have been proposed to fulfill a photoprotective role (Libkind et al. 2004). These compounds accumulate in a wide range of microorganisms that are exposed to high light intensities (Oren and Gunde Cimerman 2007), including *X. dendrorhous*, which produces mycosporines constitutively (Libkind et al. 2011). There is a direct correlation between the mycosporine content and the in situ radiation levels in many locations worldwide and in a wide variety of organisms (Oren and Gunde Cimerman 2007). However, despite our expectations, the *X. dendrorhous* ANCH isolates did not produce mycosporines under the studied conditions. Related to this, a rapid screening method for *X. dendrorhous* identification has been recently proposed that is based on simultaneous mycosporine and astaxanthin detection among red yeast isolates (Tognetti et al. 2013). Although this screening method has various advantages, especially because it is easy and fast, based in our results and on analyses performed in other wild type *X. dendrorhous* strains from collections, mycosporine production is not determinative of *X. dendrorhous* strains. Moreover, ANCH06 and ANCH08 produce low quantities of astaxanthin and could have been excluded by this analysis, though their rDNA sequences had significant similarity with *X. dendrorhous*/*P. rhodozyma*. Although the ANCH isolates do not produce mycosporines, ANCH06 had greater survival after exposure to UV-B in relation to the other tested strains. Therefore, other photoprotective mechanisms, including DNA repair mechanisms such as photoreactivation and nucleotide excision repair (Prakash and Prakash 2000; Sancar 2003), may be operating and/or enhanced in this isolate.

Ergosterol is a common membrane lipid in fungi that helps control the fluidity of membranes (Shobayashi et al. 2005) and it has been suggested that ergosterol is essential for growth at low temperatures (Hemmi et al. 1995). However, no differences between the *X. dendrorhous* Antarctic isolates and strains isolated from other regions were observed, except for ANCH06, which produces threefold less sterol than do the other strains. Therefore, we were not able to find a relationship between ergosterol content and the area in which the studied yeasts were isolated, consistent with previous reports (Tronchoni et al. 2012). Conversely, the syntheses of ergosterol and astaxanthin derive from isopentenyl-pyrophosphate, which is synthesized by the mevalonate pathway in *X. dendrorhous* (Schmidt et al. 2011). When the biosynthesis of ergosterol is blocked in *X. dendrorhous*, there is an increase in the carotenoid content and, consequently, astaxanthin (Loto et al. 2012). Additionally, the overproduction of astaxanthin by a *P. rhodozyma* mutant strain that was obtained by random chemical mutagenesis produces lower levels of ergosterol than the parental strain (Miao et al. 2011). Moreover, when a wild-type *P. rhodozyma* strain was treated with fluconazole (an ergosterol pathway inhibitor), the astaxanthin content was approximately fivefold higher than the control (Miao et al. 2011). These observations suggest that ergosterol regulates the synthesis of carotenoids by a negative feedback mechanism (Loto et al. 2012) so; high ergosterol content could be counterproductive with carotenoid biosynthesis.

As an acclimation and/or adaptation response, organisms may experience changes in their lipid composition to regulate the fluidity of cell membranes at low temperatures. For example, the proportion of unsaturated FAs (MUFAs and PUFAs) is one of the most studied responses (Rossi et al. 2009). The unsaturated FA proportion found in the ANCH isolates is similar to data from other Antarctic yeasts (Zlatanov et al. 2001). In the ANCH isolates and in UCD 67-385, these FAs are the most abundant and make up approximately 65–80 % of the total lipid composition; however, these FAs only represent about 43 % in AVHN2. The proportion of PUFAs alone make up about 14 % in UCD 67-385, but in the ANCH isolates, PUFAs are approximately twofold higher (32–37 %). This result is consistent with other reports indicating that Antarctic yeasts produce higher proportions of PUFAs (Thomas-Hall and Watson 2002). In most fungi and yeasts, palmitic, stearic, oleic, linoleic and α- or γ-linolenic acid are the most abundant FAs (Radwan 1991). These FAs were identified in all of the strains studied in this work. In the ANCH isolates, the most abundant FAs were linoleic and oleic acids, followed by palmitic and stearic acids, and they were present in proportions similar to *Cryptococcus* sp. isolated from Antarctica (Vishniac and Kurtzman 1992). Additionally, these FAs have also been identified in other *X. dendrorhous* wild type strains (Libkind et al. 2008; Sanderson and Jolly 1994), but the reported PUFA levels were higher (39 and 70.5 %) in the *X. dendrorhous* strains isolated from the Argentinean Patagonia (Libkind et al. 2008).

The ANCH01 isolate has an intense red pigmentation, which is not common in *X. dendrorhous* wild type strains. This pigmentation occurs because of the high astaxanthin yield, one of the highest amounts of astaxanthin produced by a *X. dendrorhous* wild type strain which usually is 200–400 μg g^−1^ dry yeast under the studied conditions (Schmidt et al. 2011). Considering the stressful environmental conditions from which this strain was isolated, the high production of astaxanthin is consistent with the photoprotective role of carotenoids (Schroeder and Johnson 1993). Conversely, ANCH06 and ANCH08 have a different carotenoid composition compared to other wild type strains (Andrewes et al. 1976; Schmidt et al. 2011). Despite their yellow pigmentation, these isolates produce astaxanthin, which was enhanced by random mutagenesis.

To date, *X. dendrorhous*/*P. rhodozyma* strains have been isolated from cold areas ranging from Alaska, Finland, Japan, Russia, United States and Western Europe, and recently from the Argentinean Patagonia and South Chile (Kurtzman et al. 2011). To the best of our knowledge, this work is the first report that describes the isolation and characterization of *X. dendrorhous* isolated from Antarctic soil samples. In addition, the carotenoid content and composition of the Antarctic *X. dendrorhous* isolates differs from other wild type strains because ANCH01 is a natural astaxanthin over-producer.

## Electronic supplementary material

Below is the link to the electronic supplementary material.

**Phylogenetic analysis of different** ***X. dendrorhous*** **isolates.** The consensus tree was based on ITS (ITS1, 5.8S rDNA, and ITS2) nucleotide sequence alignments and it was generated via a maximum parsimony analysis. Numbers on the branches indicate the bootstrap percentage values (1,000 replicates; values below 50% are not shown). The GenBank accession number of each sequence is indicated between brackets. The *Cryptococcus aerius* ITS sequence was used as an out-group. (PNG 8 kb)
