# Supplementary figures and images for: Identification and analysis of metabolite production with biotechnological potential in Xanthophyllomyces dendrorhous isolates
Source: World J Microbiol Biotechnol. 2015 Feb 3;31(3):517–26. doi: 10.1007/s11274-015-1808-3 (PMC4333312; doi:10.1007/s11274-015-1808-3)

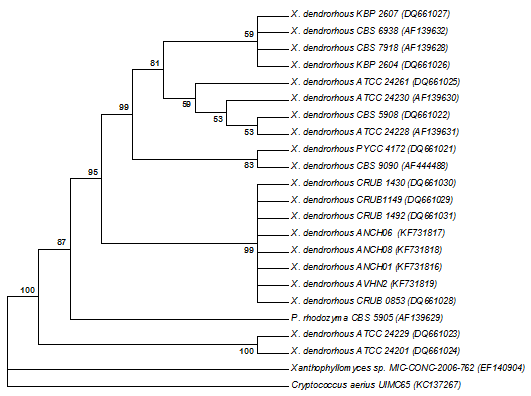

Supplement: Supplementary file 1 — Phylogenetic analysis of different X. dendrorhous isolates. The consensus tree was based on ITS (ITS1, 5.8S rDNA, and ITS2) nucleotide sequence alignments and it was generated via a maximum parsimony analysis. Numbers on the branches indicate the bootstrap percentage values (1,000 replicates; values below 50% are not shown). The GenBank accession number of each sequence is indicated between brackets. The Cryptococcus aerius ITS sequence was used as an out-group. (PNG 8 kb) [file 11274_2015_1808_MOESM1_ESM.png]
